# Pegylated liposomal doxorubicin and gemcitabine in the front-line treatment of recurrent/metastatic breast cancer: a multicentre phase II study

**DOI:** 10.1038/sj.bjc.6604409

**Published:** 2008-05-20

**Authors:** V Adamo, V Lorusso, R Rossello, B Adamo, G Ferraro, D Lorusso, G Condemi, D Priolo, L Di Lullo, A Paglia, S Pisconti, G Scambia, G Ferrandina

**Affiliations:** 1 Medical Oncology, Department of Human Pathology, University Hospital G Martino, Messina, Italy; 2 Medical Oncology, Hospital V Fazzi, Lecce, Italy; 3 Department of Gynecology/Obstetrics, Catholic University of Rome, Rome, Italy; 4 Medical Oncology, Hospital of Siderno; 5 Medical Oncology, Hospital San Vincenzo, Taormina; 6 Medical Oncology, ASL2, Isernia; 7 Gynecologic Oncology Unit, Catholic University of Campobasso, Campobasso, Italy; 8 Oncology Unit, Taranto Hospital, Taranto

**Keywords:** metastatic breast cancer, pegylated liposomal doxorubicin, gemcitabine

## Abstract

This multicentre phase II study was aimed at investigating the activity and safety of pegylated liposomal doxorubicin (PLD) and gemcitabine (GEM) as front-line therapy in a large series of chemotherapy-naïve recurrent/metastatic breast cancer patients. From June 2003 to December 2006, a total of 71 recurrent/metastatic breast cancer patients were enrolled. Median age was 63 years (range=37–79), and 31 patients (43.7%) were ⩾65 years old. Patients received PLD, 25 mg m^−2^, day 1, followed by GEM, 800 mg m^−2^, days 1 and 8, q21. Response was evaluable in 64 cases. Eight complete (12.5%) and 17 partial responses (26.6%) were registered, with an overall response rate of 39.1%. Thirty patients (46.9%) experienced stable disease, with an overall clinical benefit of 85.9%. Median time to progression (TTP) was 11 months, whereas median overall survival (OS) was not reached. The rate of 1- and 2-year OS was 79 and 61%, respectively. A total of 443 courses were evaluable for toxicity: grade 3 and 4 neutropaenia affected 14 patients (20.3%) and 3 patients (4.3%), respectively. Grade 3 and 4 palmar-plantar erythrodysesthesia syndrome was documented in five cases (7.2%) and one case (1.4%), whereas grade 3 and 4 mucositis occurred in six cases (8.7%) and two cases (2.9%), respectively. Grade 2 cardiac toxicity was observed in only one case. Interestingly enough, there was no difference in the percentage and severity of either haematological or non-haematological toxicity according to the age of the patients (<65 *vs* ⩾65 years). We confirmed in a large, very homogenous study sample of chemotherapy-naïve recurrent/metastatic breast cancer patients the efficacy and safety of PLD/GEM combination, providing response rates, median TTP and OS values comparable with those achieved with more toxic combinations.

Breast carcinoma is the most frequent cancer, and the second most leading cause of death from cancer in women (Jemal *et al*, 2007). Approximately 27% of patients are initially diagnosed with already metastatic disease, and almost 30% of lymph node-negative and up to 60–70% of lymph node-positive cases will develop recurrent disease within 5 years from initial diagnosis (Paridaens, 2000; Jemal *et al*, 2007) In this clinical setting, the prognosis is unfavourable, with a median overall survival (OS) of 1.5–2 years (Cardoso *et al*, 2002). Therefore, special attention has to be paid to the issue of quality of life preservation, as prolongation of survival and palliation of symptoms remain the only realistic objectives.

Among the drugs or drug combinations currently proposed for recurrent/metastatic breast cancer, anthracyclines and taxanes are considered the most effective ones, with rates of overall response between 46 and 88% (Mavroudis *et al*, 2000; Morales *et al*, 2004; Gamucci *et al*, 2007; Von Minckwitz, 2007); however, the use of anthracyclines in clinical practice is limited by drug-associated toxicity, particularly myelosuppression and cardiotoxicity (Robert *et al*, 2004). Indeed, the replacement of doxorubicin with epirubicin or pegylated liposomal doxorubicin (PLD) has strongly improved the pattern and severity of adverse effects (Robert *et al*, 2004; O'Brien, 2008); moreover, also the proper use of growth factor support (Morales *et al*, 2004) or the adoption of weekly schedules for combinations of anthracyclines/taxanes (Gamucci *et al*, 2007) has been proposed to overcome haematological toxicity.

Besides PLD, whose activity as a single agent in recurrent/metastatic breast cancer has been documented in several phase II studies (response rate=31–38%) (Ranson *et al*, 1997; Lyass *et al*, 2000; O'Brien *et al*, 2004), gemcitabine (GEM) has also been reported to provide encouraging response rates (range=25–37%) in this clinical setting (Carmichael *et al*, 1995; Blackstein *et al*, 2002). Moreover, the different mechanisms of action of the two drugs, which have been shown to synergise in *in vitro* and *in vivo* models (Chow *et al*, 2000; Gallo *et al*, 2006), as well as the non-overlapping toxicity profiles, further strengthened the rationale for their combination. Indeed, combined administration of PLD/GEM has been successfully investigated in ovarian cancer (Ferrandina *et al*, 2005), as well as in recurrent/metastatic breast cancer patients; in particular, in the latter subset of patients, an overall response rate between 26 and 52% (median duration of response=5.5–7 months), with an acceptable toxicity profile, has been reported in three phase II studies (Rivera *et al*, 2003; Fabi *et al*, 2006; Ulrich-Pur *et al*, 2007). However, the relatively small study samples, as well as the heterogeneity of the series, which often included a discrete proportion of patients receiving PLD/GEM combination as second line or even third or fourth line of treatment, might limit the reliability of comparison across the studies and hamper the value of data on the efficacy and the overall toxicity.

The aim of this study was to investigate the activity and safety of PLD/GEM combination as front-line therapy in a large, homogenous series of recurrent/metastatic breast cancer patients. The evaluation of response according to oestrogen (ER) and progesterone (PR) hormone receptors, as well as erbB2/neu status, has also been studied. Although trastuzumab is the indicated therapy for erbB2/neu-positive cases, we did not consider the addition of this drug to the combination, given the main objective of the study; indeed, the inclusion of trastuzumab in the subgroup of erbB2/neu-positive patients would have made the analysis of the data difficult, especially considering the underlying higher susceptibility of erbB2/neu-positive tumours to both anthracyclines and trastuzumab.

## MATERIALS AND METHODS

### Study design

This is a multicentre phase II study aimed at evaluating the activity of the combination of PLD and GEM as first-line treatment of chemotherapy-naïve recurrent/metastatic breast cancer patients.

The primary end point was the assessment of PLD/GEM efficacy in terms of clinical response and time to progression (TTP). Overall survival and the safety and tolerability of the combination were also investigated as secondary end points. The approval of the local ethic committee was obtained before the start of the trial.

### Eligibility

Patients with histologically/cytologically documented recurrent/metastatic breast cancer who had not been previously treated for metastatic/recurrent disease were enrolled. Only cases with radiological evidence of measurable (>2 cm) or evaluable disease lesions were eligible for the study. Previous adjuvant and/or neoadjuvant chemotherapy was allowed if completed >1 year before the inclusion in the study: in particular, previous treatment with anthracyclines was permitted if the cumulative dose did not exceed 350 mg m^−2^ for adriamycin, 450 mg m^−2^ for epirubicin, and 75 mg m^−2^ for mitoxanthrone. Further entry criteria were age 18–75 years, Eastern Cooperative Oncology Group performance status ⩽2, life expectancy >3 months, absolute neutrophil count (ANC) ⩾1500 *μ*l^−1^, platelet (PLT) count >100 000 *μ*l^−1^, haemoglobin levels >10 g%, bilirubin and creatinine levels less than 1.5 times the upper limit of normal, normal cardiac function (left ventricular ejection fraction (LVEF) ⩾50%), normal respiratory function, and alkaline phosphatase ⩽2.5-fold the upper normal limit. All patients were required to provide a written informed consent.

Exclusion criteria were as follows: previous or concurrent malignancies at other sites with the exception of basal or squamous cell carcinoma of the skin and cone biopsed carcinoma *in situ* of the uterine cervix; Brenner's and borderline ovarian tumours; symptomatic CNS metastases; and uncontrolled severe infection and/or medical problems unrelated to malignancy that would limit full compliance with the study or expose the patient to extreme risk. Additional exclusion criteria were as follows: previous chemotherapy with PLD or GEM, and administration of other investigational cytotoxic drugs within 30 days before entry into the study.

### Treatment plan

Within 14 days from the beginning of the study treatment, patients were submitted to a complete clinical evaluation including medical history, laboratory tests with complete blood cell count, and serum chemistry, Ca 15-3 level, and urinalysis. Bone scintigraphy, CT scan, and chest X-rays were also performed. Then, PLD, 25 mg m^−2^, diluted in 250 ml of 5% dextrose, was administered on day 1 by a 60 min i.v. infusion, followed by GEM, 800 mg m^−2^, diluted in 250 ml of 0.9% saline solution, on days 1 and 8 by a 30 min i.v. infusion; cycles were repeated every 21 days, until progression of disease, unacceptable toxicity, patients' refusal, or at pysician's discretion. All patients received dexamethasone (8 mg) and ranitidine (50 mg) before drug administration.

### Response and toxicity assessment

The evaluation of response was performed every three cycles by the same clinical and imaging approach used for the baseline assessment. Clinical response was assessed according to the RECIST criteria (Therasse *et al*, 2000). Response rates were calculated, including 95% confidence intervals (95% CI). Clinical benefit was defined as the overall number of complete responses, partial responses, and stabilisation of disease. Patients who received at least two cycles of combination treatment were evaluable for efficacy, and patients who received at least one dose of chemotherapy were evaluable for toxicity.

Chemotherapy-induced toxicity was graded according to the common toxicity criteria of National Cancer Institute (1999). Complete blood count and PLT count were performed on a weekly basis; ECG was performed at every cycle, whereas echocardiography was performed every 2 cycles and at the end of the treatment. A multigated angiogram was planned if the echocardiography registered an LVEF reduction >10% (D'Agostino *et al*, 2003). A 25% dose reduction of both drugs was planned in the case of ANC <500 *μ*l^−1^ and/or PLT count <25 000 *μ*l^−1^ on the day of planned drug administration. A 25% dose reduction of PLD was also planned in the case of grade 1 or 2 palmar-plantar erythrodysesthesia syndrome (PPE) or mucositis persisting for >2 weeks. In the case of ANC <1500 *μ*l^−1^ and/or PLT count <100 000 *μ*l^−1^ on the day of planned therapy, 1 week delay of drug administration was planned without dose adjustment. Gemcitabine administration on day 8 was omitted in the case of G4 neutropaenia, febrile neutropaenia, G3/G4 thrombocytopaenia or anaemia, or G3/G4 non-haematological toxicity (excluding alopecia or nausea/vomiting).

To reliably assess the safety of PLD/GEM combination, the prophylactic use of growth factors was not allowed, and granulocyte-colony stimulating factor (G-CSF) and/or epoetin were administered in the therapeutic setting only in the case of febrile neutropaenia or grade 3–4 neutropaenia lasting >5 days, haemoglobin levels <10 g% (Rizzo *et al*, 2002), or at the physician's discretion. Pyridoxine was not used as a prophylactic strategy to prevent PPE.

### Criteria for treatment discontinuation

In patients who had to delay treatment for >2 weeks, treatment was discontinued. Treatment was also discontinued in the case of severe hypersensitivity reaction, grade 3 or 4 PPE or mucositis persisting for >2 weeks, reduction of LVEF >20% from baseline value, in the case of symptomatic congestive heart failure, or in any case of >G3 non-haematological toxicity (with the exception of alopecia or nausea/vomiting). Treatment was also discontinued in the case of patient's refusal.

### Statistical analysis

The sample size was calculated on the basis of the two-stage design by Simon (1989). The design tested the null hypothesis that the true response rate for this population would improve by approximately 40%, that is, from 30% to the clinically relevant alternative of 50%, using an *α* error of 0.05 and a *β* error of 0.1. Thus, the first step will include 24 patients; if >8 responses are recorded, the study would enrol additional 39 patients up to a total of 63 patients. The regimen would be considered active if >24 responses are recorded. Considering a dropout rate of approximately 10%, at least 70 patients were planned to be enrolled. Overall survival was defined as time elapsed between start of PLD/GEM treatment and date of death or the date last seen. Time to progression was defined as the time elapsed between start of PLD/GEM treatment and documentation of progressive disease or the last seen. Median and life tables were computed using the product-limit estimate of Kaplan and Meyer (1958).

## RESULTS

### Patient characteristics

From June 2003 to December 2006, a total of 71 recurrent/metastatic breast cancer patients were enrolled into this phase II multicentre clinical trial.

Patient characteristics at study entry are given in Table 1. Median age was 63 years (range=37–79), and 31 patients (43.7%) were ⩾65 years old.

Forty-one (57.7%) patients had been treated with adjuvant radiotherapy, 41 patients had received adjuvant hormone therapy, and 49 (69.0%) had been administered neoadjuvant/adjuvant chemotherapy: overall, at the time of study entry, 29 (40.8%) had already been treated with anthracycline-containing regimens. The vast majority of cases (*n*=62, 87.3%) had visceral localisation of recurrent/metastatic disease, including 6 cases of liver metastasis, 7 cases of lung metastasis, and 49 cases with multiple sites of disease. The expression of ER, PR, and erbB2/neu was available in 67 cases: ER or PR positivity was documented in 43 (64.2%) and 37 (55.2%) cases, respectively, whereas 35 patients (52.2%) had erbB2/neu-overexpressing (2+/3+ score) tumours (see Table 1).

### Response to treatment and clinical outcome

Evaluation of response according to the intent-to-treat analysis and assessable population is summarised in Table 2. Seven patients were not considered evaluable for response due to early progression (*n*=2), death from non-cancer-related causes (*n*=2), severe allergic reaction during the first administration of PLD (*n*=2), and patient refusal (*n*=1). Therefore, at the time of analysis, response was evaluable in 64 cases: 8 complete responses (12.5%) and 17 partial responses (26.6%) have been registered, with an overall response rate of 39.1%. The median duration of response was 4.7 months (range=2.0–8.0), and among the responders, 6 had response lasting more than 6 months. Thirty patients (46.9%) experienced stabilisation of disease, with a rate of overall clinical benefit (complete responses, partial responses, and stabilisation of disease) of 85.9%. The median duration of stable disease and clinical benefit was 2.5 months (range=1.5–5.2) and 4 months (range=2–11), respectively.

The proportion of responders according to the site (visceral *vs* not visceral localisation) of disease was not statistically significant (60.0 *vs* 75.0%, *P*-value=0.6). In addition, there was no difference in the rate of overall response in patients who had already been treated with anthracyclines *vs* anthracycline patients (38.5 *vs* 42.4%, *P*-value=0.7).

The percentage of cases achieving complete or partial response to treatment was significantly higher in cases showing immunohistochemically assessed overexpression of erbB2/neu than in cases that do not express or express 1+ erbB2/neu (51.4 *vs* 24.1%, *P*-value=0.039). On the other hand, no difference in the response rate according to ER and PR status was documented (data not shown).

**Figure 1:**
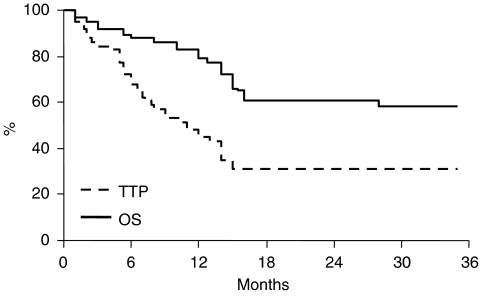
Time to progression and OS curves in the whole population.

Follow-up data were available for all patients. As of November 2007, median follow-up duration was 15 months (range=1–44). During the follow-up period, progression and death of disease were observed in 35 and 23 cases, respectively. Median TTP was 11 months, whereas the median OS was not reached (Figure 1). The rate of 1- and 2-year OS was 79 and 61%, respectively.

### Toxicity

A total of 443 courses were evaluable for toxicity, with a median number of 6 cycles (range=1–17) administered per patient (Table 3); 17 patients (24.6%) received >8 cycles of treatment.

The data on toxicity were available in 69 patients, as in 2 cases chemotherapy administration had to be discontinued early owing to the occurrence of severe allergic reaction to PLD during the administration of the first treatment course. In particular, both patients experienced sudden occurrence of cutaneous erythema (face and hands), arterial hypertension, dyspnea, and tachycardia after a few minutes since the beginning of PLD; the infusion was stopped and the patients were treated with steroids and H2-antihistamines with the regression of the described symptoms. Both patients refused to continue the treatment.

The median cumulative dose of PLD per patient was 227 mg m^−2^ (range=36–630 mg m^−2^), whereas the median cumulative dose of GEM per patient was 13 600 mg m^−2^ (range=1200–36 800 mg m^−2^) (Table 3). The delivered dose of PLD and GEM was 94 and 85% of the projected dose, respectively.

In 10 patients (14.5%), dose reduction was required, mostly because of haematological toxicity; 1 week delay was necessary in 10 patients (14.5%). There were six patients discontinuing treatment because of chemotherapy toxicity, including five cases of grade 3 PPE and one case of grade 2 cardiotoxicity.

As far as haematological toxicity is concerned (Table 4), myelosuppression was usually brief and manageable with dose adjustments or treatment delay. Grade 3 and 4 neutropaenia affected 14 (20.3%) and 3 (4.3%) patients; only 1 (1.4%) case of febrile neutropaenia was registered. Eight (11.6%) required administration of G-CSF.

Grade 3 anaemia and grade 3 thrombocytopaenia were documented in only three cases (4.3%) and one case (1.4%), respectively. Recombinant human erythropoietin (rHu-Epo) was administered to six patients (8.7%) As far as non-haematological toxicity is concerned, grade 3 asthenia was registered in only four patients (5.8%) and nausea/vomiting (grade 3) occurred in one patient.

Hepatotoxicity was infrequent and mild (grade 1 or 2=10.1%). Moderate and severe PPE was documented in five cases (7.2%) and 1 case (1.4%), respectively, whereas grade 3 and 4 mucositis occurred in six cases (8.7%) and two cases (2.9%), respectively. Complete alopecia was documented in only one case.

Although our series included 29 cases (40.8%) who had been previously treated with anthracyclines and 13 cases who were ⩾65 years old and had received previous radiotherapy to the chest, grade 2 cardiac toxicity was observed in only one case: in particular, this patient was 70 years old and had already received radiation to the left chest wall and previous treatment with anthracyclines. A reduction of 20% of LVEF from baseline value was registered after three cycles of PLD treatment (cumulative PLD dose=105 mg m^−2^), and the treatment was interrupted because of patient's refusal. Interestingly enough, there was no difference in the percentage and severity of either haematological or non-haematological toxicity according to the age of the patients (<65 *vs* ⩾65 years) (data not shown).

## DISCUSSION

We confirmed in a large, very homogenous study sample the efficacy and safety of PLD/GEM combination in chemotherapy-naïve recurrent/metastatic breast cancer patients. In particular, we showed that PLD/GEM combination provides an overall response rate of 39.1%, which is in the range of results reported by previous studies (Rivera *et al*, 2003; Fabi *et al*, 2006; Ulrich-Pur *et al*, 2007). Moreover, we reported the achievement of disease stabilisation in 46.9% of cases for an overall clinical benefit of almost 86%, which currently represents the best figure obtained with this regimen across the available phase II studies.

As previously reported (Rivera *et al*, 2003; Fabi *et al*, 2006), the overall response rate in our study did not vary according to previous exposure to anthracyclines or disease sites, whereas it was significantly higher in cases whose tumours overexpress erbB2/neu, as compared with erbB2/neu-negative cases. Amplification and/or overexpression of erbB2/neu has been recognised as a marker of susceptibility to treatment with anthracyclines (Moliterni *et al*, 2003), although several observations argue against a direct role for erbB2/neu alterations in anthracycline sensitivity (Sledge, 2001); in particular, it has been speculated that the role of erbB2/neu overexpression as a predictor of response to anthracyclines might reflect the close location of erbB2/neu and the gene coding for topoisomerase II*α*, which is the molecular target of topoisomerase II inhibitors, such as anthracyclines, and has already been shown to be involved in anthracycline susceptibility (Trost Jorgensen *et al*, 2007). Co-amplification of erbB2/neu and topoisomerase II*α* has recently been suggested to be able to define a subgroup of high-risk breast cancer patients who benefit the most from anthracycline treatment (Tanner *et al*, 2006). In this context, the most important clinical impact of our findings rely on the potential improvement of response rate and clinical outcome, deriving from selection of patients to be triaged to PLD-based regimens on the basis of concomitant assessment of erbB2/neu and topoisomerase II*α*.

With the limits inherent to the comparison across non-randomised phase II studies, our data on median TTP and OS compare well with the results obtained using other drug combinations including PLD (Addeo *et al*, 2007) or with epirubicin/taxane regimens (Mavroudis *et al*, 2000; Morales *et al*, 2004; Gamucci *et al*, 2007; Von Minckwitz, 2007). These data become even more relevant considering the favourable toxicity profile of this regimen in comparison with epirubicin/docetaxel combinations: indeed the profile of haematological toxicity was quite acceptable with percentages of moderate/severe neutropaenia lower than those reported by other authors, despite the use of G-CSF in a relatively low proportion of cases compared with trials that utilised prophylactic growth factor support (Morales *et al*, 2004).

On the other hand, we found a slightly higher percentage of mucositis/PPE than that reported with the same regimen; these findings could be related to the decision not to pre-medicate or treat PPE with pyridoxine: moreover, it cannot be excluded that the high proportion of mucositis/PPE, whose incidence and severity parallel the administered cumulative drug dose, might be also sustained by our strategy of continuing treatment until progression, which led to administer >8 cycles of PLD/GEM in 24.6% of cases.

Moreover, we observed only one case of cardiac toxicity, despite the fact that 41% of patients had already been treated with anthracyclines, and approximately 30% were ⩾65 years old; in addition, 41.9% had also been irradiated in the adjuvant setting; these data confirm the cardiac safety of PLD-based regimens (Robert *et al*, 2004; O'Brien, 2008), and support its safe use also in anthracycline-exposed older patients. Indeed, there was neither any difference in the haematological toxicity nor in the non-haematological toxicity in patients aged ⩾65 years compared with younger ones: this is a very relevant clinical issue considering that no standard chemotherapy has been established for elderly patients with recurrent/metastatic breast cancer, and efforts are constantly made to maintain the drug combination approach (Cianfrocca and Gradishar, 2007), but not at the expense of safety.

Finally, also alopecia, a treatment-related side effect hardly tolerable in patients also required to cope with the distress of disease relapse, has been reported as complete in only one case, and this is expected to contribute to preserve patient's quality of life, as shown by Fabi *et al* (2006).

In conclusion, we showed that PLD/GEM combination is active in recurrent/metastatic breast cancer patients, providing response rates, and above all median TTP and OS values comparable with those achieved with more toxic combinations (Von Minckwitz, 2007). In particular, the very low incidence of cardiac toxicity even in older patients already irradiated and treated with anthracyclines allows one to propose to a larger subset of patients a re-challenge with this class of agents, which remain the most active drugs in the management of this disease.

Finally, the possibility to take advantage of the assessment of erbB2/neu and topoisomerase II*α* expression to select the patients most likely to benefit from PLD/GEM combination, and possibly from the addition of trastuzumab in erbB2/neu-positive cases, emphasises the need to administer chemotherapy on a patient by patient basis, an issue that becomes clinically crucial in the subset of salvage treatment.

## Figures and Tables

**Table 1:** Patient characteristics at study entry

**Characteristics**	**No. (%)**
Patients enrolled	71
Age (years) (median (range))	63 (37–79)
ECOG performance status 0/1/2	53/16/2
	
*Site of recurrence*	
Liver	6 (8.4%)
Lung	7 (9.8%)
Mixed	49 (80.3%)
Bone	5 (7.0%)
Axilla	4 (5.6%)
	
*Previous treatment*	
Adjuvant RT	41 (57.7%)
Adjuvant HT	41 (57.7%)
Adjuvant/neoadjuvant CT	49 (69.0)
Anthracycline-based CT	29 (40.8%)
	
*ER status (by immunohistochemistry)*	
Positive	44 (61.9%)
Unknown	4 (5.6%)
	
*PR status (by immunohistochemistry)*	
Positive	37 (52.1%)
Unknown	4 (5.6%)
	
*ErbB2/neu status (by immunohistochemistry)*	
Negative or 1+	32 (45.1%)
2+	14 (19.7%)^a^
3+	21 (29.6)
Unknown	4 (5.6%)

ECOG=Eastern Cooperative Oncology Group; RT=radiotherapy; HT=hormone therapy; CT=chemotherapy.

a Ten out of 14 erbB2/neu 2+ cases showed DNA amplification at FISH.

**Table 2:** Clinical response in the overall series

	**Intention to treat (*n*=71)**		**Assessable (*n*=64)**	
	**No.**	**% (95% CI)**	**No.**	**% (95% CI)**
*Response*				
Complete (CR)	8	11.3 (4.1, 18.5)	8	12.5 (4.4, 20.6)
Partial (PR)	17	23.9 (14.0, 33.8)	17	26.6 (15.7, 37.3)
				
*Overall response*	25	35.2 (2.4, 46.3)	25	39.1 (27.1, 50.9)
Stable disease (SD)	30	42.2 (30.9, 53.5)	30	46.9 (34.7, 59.1)
Progression (PD)	9	12.7 (5.1, 20.3)	9	14.1 (5.6, 22.6)
Not available	7	9.9	—	—
				
Clinical benefit (CR, PR, SD)	55	77.5 (67.8, 87.2)	55	85.9 (77.4, 94.4)
Time to response (months)(median (range))		3.0 (2.0–7.0)		
Duration of response (months) (median (range))		4.7 (2.0–8.0)		
Duration of SD (months) (median (range))		2.5 (1.5–5.2)		

Duration of clinical benefit (months) (median (range))		4 (2–11)		

**Table 3:** Study drug administration details

Total cycles administered	443
Number of cycles per patient (median (range))	6 (1–17)
Cumulative PLD dose (mg m^−2^) (median (range))	227 (36–630)
Cumulative GEM dose (mg m^−2^) (median (range))	13 600 (1200–36 800)
Patients with dose reduction (no. (%))	10 (14.5%)
Patients with treatment delay (no. (%))	10 (14.5%)
Patients discontinuing treatment owing to toxicity (no. (%))	6 (8.7%)

GEM=gemcitabine; PLD=pegylated liposomal doxorubicin.

**Table 4:** Overall toxicity (per patient) (*n*=69)

	**Grade 1**		**Grade 2**		**Grade 3**		**Grade 4**	
**Toxicity**	**No.**	**%**	**No.**	**%**	**No.**	**%**	**No.**	**%**
Leukopaenia	4	5.8	16	23.2	12	17.4	1	1.4
Neutropaenia	5	7.2	19	27.5	14	20.3	3	4.3
Anaemia	17	24.6	10	14.5	3	4.3	0	—
Thrombocytopaenia	5	7.2	4	5.8	1	1.4	0	—
Fatigue	12	17.4	9	13.0	4	5.8	0	—
Nausea/vomiting	12	17.4	8	11.6	1	1.4	0	—
Liver	4	5.8	3	4.3	0	—	0	—
PPE	4	5.8	8	11.6	5	7.2	1	1.4
Mucositis	6	8.7	11	15.9	6	8.7	2	2.9

PPE=palmar-plantar erythrodysesthesia syndrome.
